# Local Ties as Self-Reported Constraints to Internal Migration in Spain

**DOI:** 10.1007/s10680-023-09661-8

**Published:** 2023-05-10

**Authors:** Jonne A. K. Thomassen, Isabel Palomares-Linares, Viktor A. Venhorst, Clara H. Mulder

**Affiliations:** Population Research Centre, Faculty of Spatial Sciences, University of Groningen, Groningen, The Netherlands

**Keywords:** Constraints to migration, Internal migration, Immobility, Local ties, Location-specific capital, Spain

## Abstract

The internal migration literature has identified various factors that deter migration and encourage staying, but has been less concerned with people’s own reports about what makes it difficult for them to migrate or makes them want to stay. We explore factors that make it difficult to change the place of residence—from here on denoted as constraints—reported in the Spanish survey on *Attitudes and Expectations of Spatial Mobility in the Labour Force* (*N* = 3892). These constraints were uniquely asked from all respondents through an open-ended question, regardless of their migration intentions. We find that many self-reported constraints correspond to factors that have previously been associated with decreased migration propensities. In order of frequency, respondents reported ties to family and friends, ties to their residential environment, financial limitations, and ties to work as constraints to migration. Our results further show that the likelihood of mentioning ties to family and friends as constraints decreased with age, was higher for women than for men and for people who lived close to most of their social network than for those who did not. Mentioning ties to the residential environment as constraints was positively associated with being partnered, and also with living in one’s birthplace. People who were unemployed were less likely to mention ties to work and were more likely to report financial limitations as constraints than people who had a permanent contract—whereas being self-employed was positively associated with mentioning ties to the residential environment.

## Introduction

The grand narrative of modernization holds up “[a] stereotype of a hypermobile society and presumptions about the importance of [internal] migration” for individuals and societies alike (Cooke, 2011, p. 195; see also Fischer, 2002). Defined as long distance moves within a country, internal migration movements are important “for the operation of regional housing and labour markets, for regional economic and cultural convergence, and for the role of mobility in shaping individual and community well‐being” (Cooke, 2011, p. 195). Nevertheless, in recent years, internal migration rates have been declining in several countries in the developed world (Champion et al., 2018). Examples are the USA (Cooke, 2013) and Australia (Bell et al., 2018). Rates have traditionally been low in other countries, such as in Spain (Bonin et al., 2008; Módenes, 1998). Seen through the lens of modernization, these increased immobility trends imply “less flexible labour markets with implications for individual economic achievement; divergence in regional economies, cultures, and politics; and greater ties to places and communities” (Cooke, 2013, p. 673).

In order to improve the understanding of low mobility, several scholars have called for a shift in focus from migration towards practices and experiences of immobility. Such calls for attention to immobility have arisen in relation to international as well as internal migration (e.g., Arango, 2000; Cooke, 2011, 2013; Coulter et al., 2016; De Jong & Fawcett, 1981; Hammar et al, 1997; Hanson, 2005; Schewel, 2020; Stockdale & Haartsen, 2018). Especially in recent years, there has been a noticeable increase in empirical studies exploring practices and experiences of immobility (e.g., Hjälm, 2014; Mærsk et al., 2021; Stockdale et al., 2018; Thomassen, 2021).

Much of the existing literature that has aimed to identify factors that deter migration or encourage staying has been based on the use of quantifiable measures of socio-demographic characteristics, household composition, socio-economic position, and the social context or local opportunity structures (e.g., Fischer & Malmberg, 2001; Mulder & Malmberg, 2014). Some of these studies focused particularly on the factors contributing to the abandonment of moving desires (e.g., Coulter, 2013; Coulter et al., 2012; De Groot et al., 2011a, 2011b).

Additional insights can be gained from people’s own reports of why they move or stay. Self-reported motives for moving and staying may provide a more nuanced description of the deterrents of migration and their relative importance. Furthermore, they may help uncover factors that have previously been missed or underestimated (Coulter & Scott, 2015). A small but growing body of literature employs self-reported motives for migration (Gillespie & Mulder, 2020; Haartsen & Thissen, 2014; Lundholm et al., 2004; Niedomysl & Amcoff, 2011; Thomas et al., 2019; Van Leeuwen & Venhorst, 2021). This literature has shown that a surprisingly large proportion of long-distance moves are motivated by family-related reasons rather than by work- or education-related reasons.

While the existing research on self-reported motives for migration is far from abundant, research that employs self-reported motives for immobility or constraints to moving is even scarcer. Some studies we were able to trace are interview-based explorations among small numbers of research participants who could be identified as stayers (Hjälm, 2014; Stockdale et al., 2018; Thomassen, 2021). Several other studies—based on survey data—used information about the importance of fixed-category factors underlying desires or decisions to stay among specific categories of young adults (Haldimann et al., 2021; Hofstede et al., 2022; Hooijen et al., 2020; Rérat, 2016). These works have painted valuable pictures of people’s immobility experiences and uncovered some of the complexity of people’s decision-making processes. Several of these studies have suggested that family and friends are important motivations for staying and constraints to moving. However, existing studies have investigated motivations for staying or constraints to moving among specific sub-populations. Therefore, they leave unclear how frequently certain types of constraints or motivations are experienced among broader populations and how they are related to a wide variety of background characteristics.

We contribute to the (im-)mobility literature by exploring self-reported constraints to migration using the responses to an open-ended survey question among a broad study population. We employ a definition of ‘constraints’ that extends beyond the obstacles to moving for those who desire to move. This is achieved by also including obstacles to even forming a desire to moving, which may encourage staying. We address two research questions. First, we ask: What constraints to migration do respondents report, and in which frequencies? To answer this question, we present the frequencies of the four most frequently reported categories of constraints: ties to family and friends, ties to one’s residential environment, ties to work, and concerns about financial limitations; plus a residual category of ‘other responses’. Second, we ask: How is the likelihood of reporting specific types of constraints associated with background characteristics, such as gender, age, household composition, geographic proximity to the social network, and socio-economic position? To answer this question, we model the likelihood of reporting each constraint category, compared to not reporting it.

The data were derived from the Spanish survey on Attitudes and Expectations of Spatial Mobility in the Labour Force (Vidal & Busqueta, 2020, *N* = 3892). This dataset contains unique information about self-reported constraints to migration based on the survey participants’ responses to the following open-ended question: ‘What is the main reason why it would be difficult for you to change your place of residence?’ Spain embodies an interesting context for this research because it is a developed country with a traditionally rather immobile population (Módenes, 1998; Palomares-Linares & van Ham, 2020), which implies the presence of widely felt constraints that deter migration or encourage staying.

## Theoretical Framework

### Establishing the Scope of Constraints to Migration

In a conceptual article on spatial choice and behaviour, Desbarats (1983) defined a constraint as “any pressure or obstacle that produces attitude-discrepant actions” (p. 350); that is, as an obstacle to mobility for people who would prefer to move. However, migration may also be constrained by an individual’s investments in non-transferable life projects in their current place of residence (e.g., Clark et al., 2017; Fischer & Malmberg, 2001). Such factors may strengthen the individual’s attachment to this place and barre them from forming a desire to migrate. Furthermore, the emerging literature on immobility has repeatedly found that staying is an active process in an individual’s residential trajectory, which may be more or less consciously re-evaluated from time to time (Hjälm, 2014; Stockdale et al, 2018; Thomassen, 2021). This implies that staying is frequently the result of obstacles that may lead to an abandonment of one’s desire to migrate or obstacles to even forming a desire to moving and motivate staying. In line with these insights, we employ a definition of ‘constraints’ that extends beyond the obstacles to moving for those who desire to move, and also includes factors that encourage staying.

### Local Ties and Other Constraints to Migration

Significant constraints to migration can arise from the presence of location-specific capital. This is a type of human or social capital that accumulates and strengthens over time, and is not easily relocated (DaVanzo, 1981). Location-specific capital includes established social relationships and professional networks, knowledge of cultural traditions, investments in the local labour market, familiarity with the local landscape, and possession of property in the local housing market (e.g., Fischer & Malmberg, 2001; Haug, 2008; Mærsk et al., 2021; Mulder & Malmberg, 2014). Such sources of location-specific capital may create attachments to place. We therefore refer to them as *local ties* (David et al., 2010; Michaelides, 2011; Mulder & Malmberg, 2014).

Many of the self-reported constraints to migration measured in our dataset are explicitly local. We study four categories of self-reported constraints in more detail: local ties to family and other social network members, local ties to one’s residential environment, local ties to work, and a category of other, non-local constraints, namely financial limitations. For each of these categories, we discuss why they could be important in people’s (im-)mobility decisions and what each of their determinants could be.

### Local Ties to Family and Friends

Social networks are important sources of support, care, and other interactions (Campbell et al., 1986; Litwak & Szelenyi, 1969). These interactions are facilitated by geographic proximity (Hank, 2007; Rainer & Siedler, 2012). Indeed, people’s local ties to family members living in their household are associated with a decreased propensity to move (Fischer & Malmberg, 2001). This is also true of ties to other geographically proximate family members, friends, and other members of their broader social network (Belot & Ermisch, 2009; Clark et al., 2017; David et al., 2010; Dawkins, 2006; Mulder & Malmberg, 2014; Palomares-Linares & van Ham, 2017). Moreover, living close to a large family network is associated with an even greater reduction in the likelihood of moving somewhere else (Palomares-Linares et al., 2019). Due to a combination of cultural preferences and structural, institutional, and social factors (Reher, 1998), proximity to family members might play an even larger role in spatial immobility in Spain than it does in other countries (see also: de Miguel Luken, 2002). Although family and friends fulfil different roles in social networks, we had to lump together ties to family members and friends. This was because many respondents mentioned them in the same breath, and some used ambiguous expressions that could refer to family, friends, or both.

*Gender* There are several reasons to think that women attach more importance than men to living close to extended family. It has, for example, been suggested that women maintain stronger relationships with family members than men do (Rossi & Rossi, 1990). Women’s family relationships are also characterised by more frequent exchanges of support (Klein Ikkink et al., 1999). For Spain, Puga (2004) observed that compared to men, women develop more social network ties in relation to their specific productive and reproductive roles. We do not have a theoretical reason to expect substantial gender differences in reporting local ties to friends (even though there are gender differences in how friendships develop over the life course; Fischer & Oliker, 1983). Given that we group ties to family and friends together, we expect *women to be more likely than men to mention ties to family and friends as constraints to migration.* When we look at the empirical work on gender differences in ties to family and friends, a mixed picture emerges. Mulder and Malmberg (2014) found no gender differences in the extent to which local ties to family deterred migration. With regard to motives for migration, Gillespie and Mulder (2020) reported that women were more likely than men to mention moving closer to non-resident family members as a motive for migration. In contrast, they found no evidence of gender differences in the likelihood of mentioning ties to friends as a motive for migration. For Spain, Ferrer and Jiménez (2009) showed that women were more likely than men to mention family ties as a reason for moving. Women were also more likely to stay in their new place of residence for a longer period if they had moved there to be close to kin.

*Age* The importance attached to relationships with family and friends could change over the life course. Whereas friends feature prominently in the networks of young adults, family ties tend to gain in importance with increasing age (Rossi & Rossi, 1990, for family; Fisher & Oliker, 1983; Gillespie et al., 2015, for friends; Gillespie et al., 2021b, for family and friends). In Spain, older people seem to prioritise family life over work or other aspects of life (CIS, 2014). At the same time, many Spanish young adults rely on family support during the early stages of their residential and labour market careers. This reliance on family may motivate them to remain close to family until they reach a certain level of autonomy (Fuster et al., 2020). Thus, although age likely plays an important role in migration decisions, there is no clear reason to expect to observe a particular age pattern in the likelihood of citing ties to family and friends as constraints to migration.

*Household composition and geographic proximity to the social network* People’s life courses are inextricably linked to those of their household members (Elder et al., 2003). Individuals therefore tend to take into account the effects of a potential move on their social relationships (e.g., Thomassen, 2021). Some people may be apprehensive about uprooting their family members’ social lives. This may especially be true of their resident children’s social lives at school and in their neighbourhood. Individuals who live with a spouse may have to consider additional ties to family and friends, such as to in-laws or the spouse’s friends. Therefore, we may expect *people with a partner and/or resident children and/or other resident family members to be more likely to mention ties to family and friends as constraints to migration than people without family members in their household.* This expectation is also based on the frequent finding in the literature that having a partner and/or resident children is associated with a decreased propensity to migrate (e.g., Fischer & Malmberg, 2001; Mulder & Malmberg, 2014; for Spain: Recaño, 2015). Naturally, ties to non-resident family and friends are only relevant as constraints to migration if they live nearby. We therefore expect *people whose social network members mostly live close by to be more likely to mention ties to family and friends as constraints to migration than people whose social network members live far away.*

*Employment* Family and friends are known to provide people with support in precarious situations, such as help with job hunting or temporary housing (Thomassen, 2021), or financial assistance (Fuster et al., 2020). Therefore, we expect *people who are currently unemployed or have a temporary contract to be more likely to mention family and friends as constraints to migration than people with a permanent contract.*

### Local Ties to the Residential Environment

People who report having strong feelings of attachment to their residential location migrate less often than those who do not (Adams, 2016). Clark et al. (2017) attributed these feelings of place attachment to the presence of family roots, connections to the community and the neighbourhood, the number of spaces used, and satisfaction. Feelings of place attachment may thus be reported as a constraint to migration.

People may also be tied to their residential location based on the locally available amenities and services. These may include higher education institutions, hospitals and care institutions, favourable housing or labour markets, the overall ‘quality of life’, the climate, or other landscape characteristics (Graves, 1976; Maza et al., 2019). Indeed, a favourable attitude towards the residential environment and its amenities has been identified as a major reason why individuals choose to stay in certain areas of Spain that deal with high unemployment rates (Artal et al., 2015; de la Fuente, 1999).

*Age* People are less likely to migrate with increasing age (e.g., Fischer & Malmberg, 2001; Mulder & Malmberg, 2014). Among other factors, this age pattern has been attributed to the tendency of people to ‘settle down’ as their feelings of attachment to their area grow stronger over time. Moreover, adults and seniors seem to feel an affiliation to place that is much more based on their experiences and links with their place of residence than is the case for young people. By contrast, young people primarily identify with a place based on their local experiences with friends and family (Cuba & Hummon, 1993). We thus expect to see a *positive association between age and mentioning ties to the residential environment as constraints to migration.*

*Household composition and geographic proximity to social network members* Feelings of place attachment may be more pertinent for people who feel ‘settled’. Fischer and Malmberg (2001) attributed ‘settling down’ not only to age patterns, but also to local life projects, such as being married, having children, and being a homeowner. Thus, the presence of a partner, children, or other family members in the household may lead people to feel tied to their residential environment. Therefore, we expect *people who are living with a partner, children, and/or other family members to be more likely to mention ties to their residential environment as constraints to migration than people who are single, childless, or without other resident family members.* Furthermore, as place attachment is rooted in local family and neighbourhood connections (Clark et al., 2017), people with social network members living nearby may have a greater tendency to develop ties to their local environment. Thus, we expect *people whose social network members mostly live close by to be more likely to mention ties to their residential environment as constraints to migration than people whose social network members live far away.*

*Living in one’s birthplace* Living in, or near, one's birthplace is associated with a low propensity to migrate (Mulder & Malmberg, 2014). Moreover, people who have returned to their birthplace after having moved away may also have strong feelings of attachment to their place of residence. That is because they are likely to be familiar with the surroundings and to have location-specific capital there. Indeed, the special relationship many Spanish people have with their birthplace has been identified as a factor in that country’s low migration rates (Palomares-Linares, 2018) and high return migration rates (Puga, 2004). We thus expect to find that *people who no longer live in their birthplace are less likely to mention local ties to their residential environment as constraints to migration than people who live in their birthplace.*

### Local Ties to Work

Local ties to work may constrain migration, especially among people who work close to home (Mulder & Malmberg, 2014), who cannot easily change their work location, or who feel attached to their job.

*Gender* According to the gender role model of family migration (e.g., Cooke, 2013), there is reason to believe that in opposite-sex couples, ties to the work of the male partner will have a more constraining effect on a couple’s propensity to migrate than those of the female partner. We therefore expect *men to be more likely than women to mention ties to work as constraints to migration.* However, Mulder and Malmberg (2014) found no support for the idea that in opposite-sex couples, the man’s local ties to work would have a stronger association with the actual likelihood of migrating than the woman’s.

*Age* The job change hypothesis argues that over time, people find jobs that fit their needs better (Wright & Hamilton, 1978). Thus, people’s feelings towards their job may become more positive with age. Indeed, many studies have found a positive relationship among workers between age and job satisfaction (for an overview, see: Barnes-Farrell & Matthews, 2007). However, people’s work values differ between cohorts, as they are shaped by generational experiences, such as financial and security crises (Smola & Sutton, 2002). Our cross-sectional data do not allow us to distinguish between age and birth cohort. Furthermore, no direct links between age, workplace satisfaction, and migration have emerged from the literature. Therefore, we are undecided about which association we expect to observe between age and the likelihood of reporting ties to work as constraints to migration.

*Employment* Naturally, we would not expect unemployed people to feel constrained from migrating because of ties to work. Among employed people, those who have a temporary contract know that they will have to leave the company in the future. They may thus develop fewer ties to work than those with a permanent contract. It has traditionally been argued that self-employed people and entrepreneurs tend to be attached to a local clientele, and are therefore less likely than salaried workers to migrate (Koster & Venhorst, 2014). Today, however, some self-employed people work online, and may thus be less constrained to move. We expect *unemployed people and those with a temporary contract to be less likely, and self-employed people to be more likely, to mention ties to work as constraints to migration than individuals with a permanent contract.*

People’s occupations may also be associated with developing more or fewer ties to work. In the Spanish labour market, professionals are more likely than workers in other occupations to have a history of migration (Mulder et al., 2022). Conversely, managers and directors may feel more attached to their job or loyal to their place of work because they supervise teams and employees who depend on them. We therefore expect *people in other occupations to be more likely than professionals to mention ties to work as constraints to migration*.

### Financial Limitations

While migration has been characterised as a tool for coping with socio-economic and labour market vulnerability (see: Clark, 1982), people also need information and resources in order to move. Having insufficient financial resources makes it more difficult for people to cover the monetary costs of moving. These costs may include the ‘out-of-pocket expenses’ of food, lodging, and transportation (Sjaastad, 1962). Moreover, people who move may have to put down a deposit to rent a new dwelling, pay real estate agencies for their services, cover the cost of a new dwelling before their old dwelling has been sold, and cover other transaction costs. Thus, financially vulnerable people may not feel well-equipped to navigate the migration process, and might therefore mention financial limitations as constraints to migration. Indeed, Landale and Guest (1985) suggested that a lack of resources constrains people’s migration intentions from being realised.

*Age* According to Collins and Urban (2020), people’s financial well-being—measured as their subjective financial status and perceived future financial trajectory—increases with age. In Spain, high levels of labour and housing market vulnerability among young people (Fuster et al., 2019) point to an age pattern in financial well-being. Therefore, we expect* age to be negatively associated with mentioning financial limitations as constraints to migration.*

*Household composition* When partners share a household, they can pool resources. Indeed, the probability of moving has been found to increase with household income (Clark & Huang, 2003; Coulter, 2013; Coulter et al., 2011; de Groot et al., 2011a; Lu, 1998). Thus, we expect to find that *people who have a partner are less likely than single people to mention financial limitations as constraints to migration.* It is not immediately clear what effect having children in the household has on people’s migration decisions. On the one hand, this life phase tends to be associated with financial stability. On the other hand, people with children may feel a strong responsibility to support their family, and thus be less inclined to take the financial risk of moving.

*Education and employment* The distribution of earnings is largely determined by the level and the distribution of schooling (Becker & Chiswick, 1966; Mincer, 1974). Having a university education improves an individual’s human capital, and may lead to higher-paying professional and managerial jobs. We therefore expect to find that *people who have a university degree are less likely than people who do not to mention financial limitations as constraints to migration.* People who lack secure employment or who work in a lower-paying job may have insufficient resources to cover moving costs. Therefore, we expect *people who are unemployed or who have a temporary contract to be more likely to mention financial limitations as constraints to migration than people who are permanently employed or are self-employed.* Having little or no income has been found to deter people from migrating (Kley, 2011; Kley & Mulder, 2010). However, being unemployed has been shown to both decrease (de Groot et al., 2011a) and increase (Mulder & Malmberg, 2014) the propensity to migrate. We also expect to find that *professionals, managers, and directors are less likely than people in other occupations to mention financial limitations as constraints to migration*.

## Data and Methods

### Dataset and Sample

We use cross-sectional data from the Spanish survey on Attitudes and Expectations of Spatial Mobility in the Labour Force (Vidal & Busqueta, 2020)*.* The data were collected through computer-assisted web interviews in October 2019.^1^ The main objective of the survey was to explore the participants’ willingness to migrate based on their perceptions of the constraints in their current place of residence, and of opportunities elsewhere. The structured questionnaire contained 51 questions, and the primary topics covered were employment, migration history, mobility intentions, and willingness to migrate. The survey also asked respondents about their values, their life expectations, and the socio-demographic characteristics of themselves and their households. While most of the questions had fixed-category responses, questions regarding constraints to and reasons for migration were open-ended.

The target population were between 18 and 55 years old, resided in Spain, and participated in the labour force by being (self-)employed or seeking employment. The sample was stratified according to three characteristics: region of residence (eight Nielsen zones), age group (18 to 24, 25 to 39, and 40 to 55), and sex. However, as is often the case in online surveys, the response rate was low (12%). Several efforts were made to obtain a sample representative of the population of Spain, for example through quota sampling. To assess the representativeness, Mulder et al., (2022) compared the survey’s sample with the sample of the third quarter of 2019 in the Spanish Labour Force Survey. They found no relevant divergences in sample composition with regard to socio-demographic characteristics, level of education, or employment situation.

The survey yielded 4008 viable responses. We dropped the responses of people who were serving in the military (*n* = 23) because they may not have been free to choose where they lived, and thus would not have been constrained in their migration decisions by the same factors as the general population. We also dropped cases that did not contain information about one or more of the following independent variables: relationship to the respondent’s birthplace (*n* = 19 missing values), university education (*n* = 14), occupation (*n* = 22), or geographic proximity to social network members (*n* = 40). The final sample size was 3892.

### Dependent Variables

To construct the dependent variables, we used the responses to the following open-ended question: ‘What is the main reason why it would be difficult for you to change your place of residence?’ A further instruction read: “by place we mean the town/village where you live and its surroundings”.^2^ All respondents were asked this question, regardless of their moving intentions. We use the term migration to denote all these potential moves from the place of residence. Although respondents were asked to cite the ‘main reason’, 358 respondents mentioned two or three constraints. Initially, we labelled all first, second, and third constraints separately (*N* = 4289) using an inductive approach, which resulted in a primary coding scheme with keywords in English. Then, we grouped the subcategories around major themes drawn from the (im-)mobility literature, which yielded four types of constraints and a rest category. In Appendix 1, we describe the coding and operationalisation processes in more detail. Table 5 in Appendix 1 shows the reported frequencies per constraint category and per subcategory, and the associated inductive keywords.

To prevent information loss for respondents who mentioned more than one constraint (9%), we constructed four separate binary dependent variables that measured whether a particular type of constraint was reported or not. These four dependent variables indicate whether or not the respondents felt constrained by: (1) *local ties to family and friends*; (2) *local ties to the residential environment*; (3) *local ties to work*; and (4) *financial limitations*. The reference category of each variable contains all responses in which the outcome of interest was not reported, including those cases in which the respondent did not mention any constraint. In Table 1, we report the frequencies of the dependent variables. For a sensitivity analysis we also specified a categorical dependent variable based on the respondents’ first-mentioned constraint, with four categories for the four constraints and one category for other responses; results are shown in Appendix 1 (Table 6).

**Table 1:** Descriptive statistics of independent and dependent variables (*N* = 3892)

Independent variables	Column % or mean (SD)	
Sex		
Men	50.4	
Women	49.6	
Age in years	38.0 (9.8)	
Household		
Single without children	25.2	
Single with children	8.2	
Partner without children	22.3	
Partner with children	44.4	
Other resident family members		
No	79.5	
Yes	20.6	
All or most social network members live close by		
No	31.5	
Yes	68.5	
Employment status		
Permanent contract	59.1	
Temporary contract	16.2	
Self-employed and entrepreneurs	11.9	
Unemployed	12.9	
Occupation		
Professionals	20.8	
Manual workers	26.2	
Commercial, service, and administrative sector	28.9	
Managers and directors	21.6	
Never worked before	2.5	
University education		
No	53.7	
Yes	46.3	
Relationship to birthplace		
Living in the birthplace	65.4	
Not living in the birthplace	26.2	
International migrant	8.4	
Migration intention		
Not considering to migrate	55.3	
Considering to migrate	24.4	
Considering and planning to migrate	20.3	
Geographic region of residence		
North	16.7	
East	14.3	
South	24.9	
Central	16.2	
Metropolitan area: Madrid	14.4	
Metropolitan area: Barcelona	13.5	

Dependent variables	Column %	*n*
Ties to family and friends		
Mentioned	33.0	1285
Not mentioned	67.0	2607
Ties to the residential environment		
Mentioned	17.9	698
Not mentioned	82.1	3194
Ties to work or the partner’s work		
Mentioned	9.6	374
Not mentioned	90.4	3518
Financial limitations		
Mentioned	11.8	459
Not mentioned	88.2	3433

*Source*: Survey on Attitudes and Expectations of Spatial Mobility in the Labour Force (Vidal & Busqueta, 2020)

### Independent Variables

All four models included the same set of independent variables (frequencies shown in Table 1). We included *sex* as a dummy variable and *age* in years. We measured household composition with two variables: (1) a four-category variable measuring *living with or without a partner, and with or without children*; and (2) a dummy variable measuring *whether or not any parents and/or other family members were living in the household*. We measured geographic proximity to social network members as a dummy indicating whether individuals *were or were not living close to most or all of their social network members.* Furthermore, we used three indicators of socio-economic status: (1) a dummy for *university education*; (2) a four-category variable measuring *employment status* (permanent contract, temporary contract, self-employed, unemployed); and (3) a five-category variable indicating the type of *occupation* (professionals, manual workers, administrative/services workers, managers/directors, and a residual category of people who had never worked and thus could not be categorised into any occupation type). We used a three-category variable indicating *where people were living in relation to their birthplace:* living in their birthplace (including return migrants), living outside their birthplace but having been born in Spain, and international migrants living in Spain. Finally, we introduced two control variables. One categorical variable measured *migration intention*. This allowed us to take into account whether respondents were: not considering to migrate, considering to migrate, or considering and planning to migrate at the time they reported their constraints. Another control was included for the *geographic region of residence* categorised into four large zones (North-West, East, South, and Central) and the two main metropolitan areas of Spain (Madrid and Barcelona). Measures of homeownership and household income were also included in the dataset. However, 30% of these two variables contained missing values. The missing values on homeownership were surprising, but they proved to be uncorrelated with all other variables in the dataset. They did follow a similar pattern as those on income. We thus excluded both variables from the analyses in order to not bias our results.

### Analytical Approach

We ran separate binary logistic regressions for each of the four outcomes. We preferred this approach over one multinomial model because it prevents information loss for the 9% of the respondents who mentioned more than one constraint. The sensitivity check using one multinomial regression model allows for comparisons between outcome categories, but can only take into account the first constraint mentioned (results shown in Appendix 1, Table 6). The findings are very similar to those from the separate logistic regression models. We present the models using unweighted data. A sensitivity check using weighted data (tables available upon request) revealed that the results were similar to those of the unweighted models.

## Results

### Frequencies of Reporting the Constraints

**Fig. 1:**
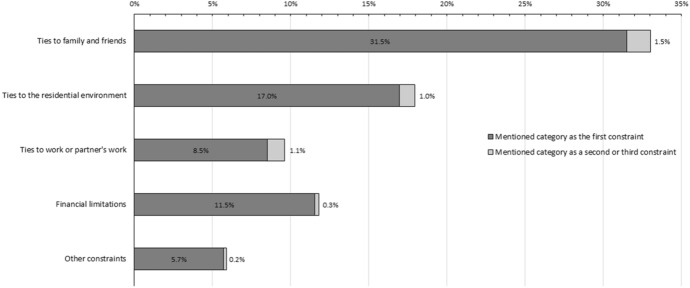
Percentage of respondents (*N* = 3892) mentioning each category of self-reported constraint to migration as the first constraint, or as a second or third constraint. *Note*: 25.8% of respondents did not mention any constraint. *Source*: Survey on Attitudes and Expectations of Spatial Mobility in the Labour Force (Vidal & Busqueta, 2020), authors’ calculations

The constraint category that was most frequently cited as the first constraint (31.5% of the respondents) and as a second or third constraint (1.5%) was that of local ties to family and friends (Fig. 1). Respondents also frequently reported ties to the residential environment (17.0%), ties to work (8.5%), and concerns about financial limitations (11.5%) as the first constraint. Of those three categories, financial limitations were least frequently mentioned as a second or third constraint.

Table 2 shows that there were large differences by socio-demographic characteristics in how frequently specific constraints were mentioned. For example, ties to family and friends were mentioned particularly frequently (> 35%) by women, people who were living with family members other than their partner or children, and people whose social network members were living close by.

**Table 2:** Descriptive findings for mentioning specific constraints conditional on the values of the independent variables: percentage reported and *p* of Chi-square test or mean (SD) and *F*-test (*N* = 3892)

	Dependent variables				Other responses	
	Ties to family and friends	Ties to the residential environment	Ties to work or partner’s work	Financial limitations	Other answers	No answer
	% Reported or mean (SD)	% Reported or mean (SD)	% Reported or mean (SD)	% Reported or mean (SD)	% Reported or mean (SD)	% Reported or mean (SD)
Sex						
Men	30.2	18.1	9.7	13.6	6.7	25.8
Women	35.9	17.7	9.5	10.0	5.0	25.8
*p*(Chi2)	*0.000*	*0.730*	*0.874*	*0.001*	*0.024*	*0.993*
Age						
Mean age in years	37.8 (9.5)	39.1 (9.7)	39.5 (9.6)	37.8 (10.1)	37.4 (10.7)	37.5 (9.7)
*F-test(p)*	*1.2 (0.277)*	*9.7 (0.002)*	*9.1 (0.003)*	*0.4 (0.506)*	*1.1 (0.290)*	*4.5 (0.035)*
Household						
Single without children	33.5	14.8	7.8	15.1	7.8	24.3
Single with children	33.7	12.9	9.1	10.7	3.1	33.7
Partner without children	29.1	18.9	10.6	11.1	5.6	28.8
Partner with children	34.6	20.2	10.3	10.5	5.5	23.7
*p*(Chi2)	*0.044*	*0.000*	*0.126*	*0.003*	*0.010*	*0.000*
Other resident family members						
No	32.2	18.6	10.5	10.7	6.0	26.1
Yes	36.0	15.4	6.4	15.9	5.6	24.5
*p*(Chi2)	*0.044*	*0.034*	*0.000*	*0.000*	*0.727*	*0.347*
All or most social network members live close by						
No	28.1	17.4	12.3	14.4	6.7	25.4
Yes	35.3	18.2	8.4	10.6	5.5	26.0
*p*(Chi2)	*0.000*	*0.536*	*0.000*	*0.001*	*0.148*	*0.678*
Employment status						
Permanent contract	34.3	17.9	10.7	10.5	5.8	25.1
Temporary contract	35.0	17.5	7.6	13.7	4.3	25.1
Self-employed and entrepreneurs	23.4	20.6	10.0	9.1	7.8	33.1
Unemployed	33.7	16.3	7.0	17.7	6.6	23.3
*p*(Chi2)	*0.000*	*0.372*	*0.020*	*0.000*	*0.094*	*0.002*
Occupation						
Professionals	34.0	18.1	10.5	13.0	4.4	23.6
Manual workers	31.0	17.2	8.3	10.8	7.1	29.1
Commercial, service, and administrative sector	35.8	17.5	10.3	12.6	5.0	23.2
Managers and directors	32.0	19.1	8.5	10.4	6.8	27.6
Never worked before	22.2	20.2	17.2	15.2	7.1	24.2
*p*(Chi2)	*0.019*	*0.800*	*0.026*	*0.237*	*0.057*	*0.010*
University education						
No	32.6	17.9	10.0	12.2	6.6	24.8
Yes	33.5	18.0	9.2	11.4	5.1	26.9
*p*(Chi2)	*0.536*	*0.945*	*0.435*	*0.454*	*0.055*	*0.139*
Relationship to birthplace						
Living in the birthplace	34.3	18.9	8.2	10.8	5.8	26.0
Not living in the birthplace	32.9	15.9	12.5	12.8	5.8	24.0
International migrant	23.3	16.6	11.7	16.9	6.8	29.5
*p*(Chi2)	*0.000*	*0.083*	*0.000*	*0.003*	*0.786*	*0.137*
Migration intention						
Not considering to migrate	31.7	23.1	10.7	8.5	3.7	27.5
Considering to migrate	38.6	10.4	8.5	16.3	6.7	22.2
Considering and planning to mi grate	29.8	12.9	8.0	15.3	10.9	25.5
*p*(Chi2)	*0.000*	*0.000*	*0.038*	*0.000*	*0.000*	*0.008*
Geographic region of residence						
North-West	34.5	17.0	10.3	8.6	7.1	26.8
East	34.6	17.7	10.2	10.9	6.3	24.4
South	34.6	16.8	8.9	11.9	5.6	25.8
Central	34.3	19.7	8.4	11.8	6.4	24.0
Metropolitan area: Madrid	32.4	17.4	10.9	15.1	4.5	24.4
Metropolitan area: Barcelona	25.6	19.9	9.5	13.0	5.5	29.8
*p*(Chi2)	*0.006*	*0.544*	*0.661*	*0.020*	*0.481*	*0.228*

*Source*: Survey on Attitudes and Expectations of Spatial Mobility in the Labour Force (Vidal & Busqueta, 2020), authors’ calculations

### Results of the Logistic Regressions

In Table 3, we present the results of the four binary logistic regressions. We also provide an overview of the expected associations and the results for these associations in Table 4.

**Table 3:** Binary logistic regressions per category of self-reported constraints to migration (1 = reported; 0 = not reported)

	Ties to family and friends			Ties to the residential environment			Ties to work or partner’s work			Financial limitations		
	*B*	SE *B*	*p*	*B*	SE *B*	*p*	*B*	SE *B*	*p*	*B*	SE *B*	*p*
Women (ref: men)	0.269	0.072	0.000	− 0.042	0.088	0.636	− 0.038	0.114	0.739	− 0.447	0.106	0.000
Age (in years)	− 0.008	0.004	0.048	0.007	0.005	0.168	0.014	0.006	0.026	0.009	0.006	0.126
Household (ref: Single without children)												
Single with children	0.121	0.150	0.418	− 0.176	0.203	0.386	− 0.016	0.245	0.948	− 0.153	0.219	0.484
Partner without children	− 0.095	0.122	0.438	0.336	0.150	0.025	0.091	0.187	0.628	− 0.126	0.169	0.453
Partner with children	0.204	0.113	0.070	0.338	0.141	0.017	0.008	0.176	0.964	− 0.121	0.158	0.444
Resident family members (ref: no)	0.201	0.112	0.072	0.101	0.143	0.476	− 0.309	0.191	0.107	0.249	0.152	0.101
All or most social network members live close by (ref: no)	0.315	0.090	0.000	− 0.147	0.109	0.178	− 0.325	0.135	0.016	− 0.183	0.124	0.140
Employment status (ref: permanent contract)												
Temporary contract	0.003	0.101	0.975	0.112	0.126	0.372	− 0.347	0.172	0.044	0.254	0.144	0.078
Self-employed and entrepreneurs	− 0.502	0.122	0.000	0.277	0.133	0.037	− 0.075	0.175	0.669	− 0.299	0.181	0.098
Unemployed	0.027	0.117	0.819	0.002	0.149	0.987	− 0.723	0.215	0.001	0.495	0.155	0.001
Occupation (ref: professional)												
Manual workers	− 0.216	0.105	0.039	− 0.040	0.130	0.755	− 0.179	0.168	0.286	− 0.247	0.153	0.105
Commercial, service, and administrative sector	− 0.034	0.101	0.735	− 0.067	0.126	0.593	0.018	0.156	0.910	− 0.013	0.144	0.928
Managers and directors	− 0.155	0.110	0.157	0.068	0.133	0.608	− 0.276	0.175	0.113	− 0.229	0.161	0.156
Never worked before	− 0.780	0.271	0.004	0.293	0.291	0.313	1.197	0.330	0.000	− 0.183	0.328	0.577
University education (ref: no)	0.002	0.074	0.975	− 0.009	0.091	0.920	− 0.131	0.118	0.268	− 0.040	0.108	0.709
Relationship to birthplace (ref: living in the birthplace)												
Not living in the birthplace	0.085	0.090	0.349	− 0.272	0.114	0.017	0.299	0.138	0.030	0.115	0.131	0.380
International migrant	− 0.352	0.152	0.020	− 0.226	0.177	0.202	0.232	0.210	0.269	0.403	0.184	0.029
Migration intention (ref: not considering to migrate)												
Considering to migrate	0.360	0.084	0.000	− 0.943	0.120	0.000	− 0.217	0.139	0.118	0.701	0.120	0.000
Considering and planning to migrate	− 0.045	0.095	0.635	− 0.681	0.122	0.000	− 0.242	0.154	0.115	0.628	0.131	0.000
Geographic region of residence (ref: North-West)												
East	− 0.008	0.124	0.950	0.090	0.155	0.564	− 0.038	0.193	0.842	0.256	0.198	0.197
South	0.008	0.109	0.941	− 0.006	0.139	0.964	− 0.114	0.175	0.514	0.340	0.176	0.053
Central	− 0.011	0.120	0.929	0.125	0.148	0.400	− 0.260	0.196	0.185	0.420	0.191	0.027
Metropolitan area: Madrid	− 0.139	0.125	0.264	0.077	0.156	0.621	0.074	0.190	0.696	0.701	0.187	0.000
Metropolitan area: Barcelona	− 0.489	0.132	0.000	0.269	0.155	0.082	− 0.064	0.200	0.749	0.460	0.195	0.018
Constant	− 0.718	0.235	0.002	− 1.620	0.287	0.000	− 2.212	0.365	0.000	− 2.716	0.344	0.000
Model summaries												
N	3892			3892			3892			3892		
LR Chi2(*df*)	132.05 (24)			125.90 (24)			73.69 (24)			128.17 (24)		
Prob > Chi2	0.000			0.000			0.000			0.000		
Pseudo *R*^2^	0.0267			0.0344			0.0299			0.0454		

*Source*: Survey on Attitudes and Expectations of Spatial Mobility in the Labour Force (Vidal & Busqueta, 2020), authors’ calculations

**Table 4:** Summary of the expected associations between background characteristics and reporting four types of constraints to migration

Characteristic	Local ties to family and friends		Local ties to residential environment		Local ties to work		Financial limitations	
	Expected	Found	Expected	Found	Expected	Found	Expected	Found
Women (ref: Men)	+	+	None	None	−	None	None	−
Age	Undecided	−	+	None	Undecided	+	−	None
Household (ref: Single without children)								
Single with children	+	None	+	None	None	None	Undecided	None
Partner without children	+	None	+	+	None	None	−	None
Partner with children	+	+ (90%)	+	+	None	None	Undecided	None
Other resident family members (ref: No)	+	+ (90%)	+	None	None	None	None	None
Most or all social network members live close by (ref: No)	+	+	+	None	None	−	None	None
Employment status (ref: Permanent contract)								
Temporary contract	+	None	None	None	−	−	+	+ (90%)
Self-employed and entrepreneurs	None	−	None	+	+	None	None	− (90%)
Unemployed	+	None	None	None	−	−	+	+
Occupation (ref: Professionals)								
Manual workers	None	−	None	None	+	None	+	None
Commercial, service, and administrative sector	None	None	None	None	+	None	+	None
Managers and directors	None	None	None	None	+	None	None	None
Never worked before	None	−	None	None	None	+	None	None
University education (ref: No)	None	None	None	None	None	None	−	None
Relationship to birthplace (ref: Living in birthplace)								
Not living in the birthplace	None	None	−	−	None	+	None	None
International migrant	None	−	None	None	None	None	None	+

+, (more likely); −, (less likely); none (no expected or found difference); undecided (theoretical direction unclear); 90% (found difference with *p* < 0.1)

*Sex* In line with our hypothesis, women were more likely than men to mention ties to family and friends as constraints to migration (*B* = 0.269, implying an odds ratio of exp[0.269] or 1.309; *p* = 0.000). We also found that women were considerably less likely than men to mention financial limitations as migration constraints. These observed gender differences may be related to the persistence of gender roles, with women being the main caregivers and men having the bulk of the financial responsibilities. For similar reasons, we had expected to find that women would be less likely than men to mention local ties to work, but we did not find support for this hypothesis. This is in line with earlier findings by Mulder and Malmberg in Sweden (2014). Overall, the observed associations between gender and reporting specific categories of constraints may be understood as a sign that women in Spain have increasingly taken up roles in the productive sector, but their roles in the domestic and reproductive spheres have changed less quickly (Salido, 2011).

*Age* Given that location-specific capital tends to accumulate and strengthen over time (DaVanzo, 1981), we expected to find a positive association between age and the likelihood of mentioning ties to the residential environment. The positive association we found is not statistically significant, however. We were undecided about the expected associations between age and the likelihood of reporting ties to family and friends as well as ties to work. We found a negative association between age and mentioning ties to family and friends. This may relate to the finding from a qualitative work by Fuster et al. (2020) that a strong reliance on family support makes young adults in Spain want to remain close to family members. We found a positive association between age and mentioning ties to work. We cannot be certain whether this means that attachment to work increases with age, or that our cross-sectional data was picking up on differences between the labour force roles of the younger and the older generations (Smola & Sutton, 2002). The survey was collected at a time of high unemployment rates, increasing job vulnerability, and job precariousness among young Spanish people (Fuster et al, 2019). Nevertheless, we did not find support for a negative association between age and mentioning financial limitations.

*Household composition and geographic proximity to the social network* We found little variation in the likelihood of reporting specific constraints according to the respondents’ household composition. For example, we found that living with a partner and children or with family members other than a partner and children was positively associated with mentioning ties to family and friends. However, these associations were not very strong and only marginally significant. Regarding ties to the living environment, we found positive associations for those living with a partner—with or without children—compared to singles without children. We did not find any significant associations between any of the other expectations regarding the household composition and reporting ties to the living environment or financial limitations.

As expected, people whose social network members lived close by were more likely to mention ties to family and friends as constraints to migration. This finding emphasizes that family and friends who live close by are experienced as constraints, and should be understood as a motivation for immobility. This is in line with earlier findings on the role of social ties determinants for staying behaviour in Spain (Clark et al., 2017; Palomares et al., 2019) as well as other contexts (Schewel & Fransen, 2022). While we did not formulate any hypotheses about this, those whose family and friends lived close by were also less likely to report ties to work. However, we did not find support for the hypothesis that living close to the social network would also be positively associated with citing ties to the living environment.

*Employment* Surprisingly, we found that self-employed respondents were much less likely to report ties to family and friends, and marginally less likely to report financial limitations, than respondents with a permanent contract. We also unexpectedly found a positive association between reporting ties to the residential environment and being self-employed or an entrepreneur. However, we did not find evidence for the expected positive association between reporting ties to work and being self-employed or an entrepreneur. In a previous study, Koster and Venhorst (2014) found that self-employed people relocated their business more often than their residence. The authors attributed this finding to a desire to stay close to their social ties while improving the location of their business. Our findings suggest that the main constraints to moving for self-employed respondents were not their ties to family and friends, but their ties to their residential environment.

Furthermore, we found the expected negative association between having a temporary contract or being unemployed and mentioning ties to work as constraints to migration, compared to having a permanent contract. With regards to reporting financial limitations as constraints to migration, we found the expected positive associations for those with a temporary contract and those who are unemployed. However, the effect was marginally significant for those with a temporary contract. We did not find support for our hypotheses that people who had a temporary contract or were unemployed would be more likely to mention ties to family and friends than people who had a permanent contract.

*Occupation and education* We had expected to find differences in the likelihood of citing ties to work and financial limitations as constraints to migration based on people’s socio-economic position. However, our findings showed little variation based on people’s occupation and education. We only found a statistically significant, positive association for people who had never worked before in reporting ties to work, compared to professionals. We were surprised to find that, instead, manual workers and people who had never worked before were less likely than professionals to mention ties to family and friends. We see no reason to think that these individuals attached less importance to family and friends than professionals. Instead, we speculate that there may have been more respondents among the professionals (whose jobs are more likely to require migration; e.g., see Mulder et al., 2022) who have experienced living at a distance from their family and friends before. Furthermore, while we had expected to observe that people who had a university degree would be less likely to mention financial limitations than people who did not, we found no evidence for such an association.

*Migration history, migration intention, and geographic location* We find support for a negative association between reporting ties to the residential environment and no longer living in the birthplace. We also found evidence for a negative association between being an international migrant and reporting ties to family and friends. This finding is not surprising given that some or all of the family and friends of international migrants likely live abroad. In addition, we observed a statistically significant positive association between no longer living in one’s birthplace and mentioning ties to work as well as mentioning financial limitations.

Our results did not change substantially once we controlled for the respondent’s migration intentions and geographic locations. Yet, it is interesting to note that considering as well as planning to migrate was negatively associated with reporting ties to the living environment and positively associated with reporting financial limitations. It might be that considering to migrate makes people more aware of the financial resources needed to make the move, while ties to the living environment and work are important constraints for those who are not currently considering to migrate. Considering to migrate was positively associated with mentioning ties to family and friends.

## Conclusions and Discussion

With this paper, we aimed to advance the (im-)mobility literature by exploring self-reported constraints to migration. We were able to do so drawing on the answers to an open-ended question in the Spanish survey on *Attitudes and Expectations of Spatial Mobility in the Labour Force* (Vidal & Busqueta, 2020).

Our analyses of self-reported constraints to migration across a uniquely broad study population showed that a large proportion of constraints corresponded to three types of non-transferable factors, known as local ties. Previous studies have shown such ties to be associated with a decreased propensity to migrate (e.g., Fischer & Malmberg, 2001; Michaelides, 2011; Mulder & Malmberg, 2014). Our results help understand the relative importance of these constraints in people’s (im-)mobility decisions. Apparently, the constraints that people report are indeed mostly connected with ties to their local social and physical environment. Nevertheless, another category of frequently reported constraints included concerns about financial limitations. This finding points to the need for resources to undertake migration and to the role of financial investments like mortgages in constraining migration (e.g., Landale & Guest, 1985; Sjaastad, 1962). Financial limitations seemed to have materialized into a constraint to migration especially for those who were indeed considering or planning to migrate; they were much more likely to report financial constraints than those who were not currently considering to migrate.

We would like to call specific attention to the relatively high frequencies of mentioning ties to family and friends as constraints (more than one-third of the respondents). While some respondents mentioned ‘family and friends’ in one breath, others explicitly mentioned the obligation they felt towards a specific social network member (for more keywords, see: Appendix 1, Table 5). The high incidence of reporting ties to family and friends might be partly related to strong feelings of family solidarity, which are specific to contexts like Spain and other societies with collectivistic family traditions. However, the important roles of family and friends in people’s (im-)mobility decisions have also been emphasised in qualitative studies conducted in more individualistic societies (e.g., Hjälm, 2014; Stockdale et al., 2018; Thomassen, 2021). Furthermore, surprisingly large proportions of family- and friend-related responses have also been documented in studies that explored self-reported motives for migration. Such studies have been conducted in Spain (Puga, 2004), but also in North-West Europe (Gillespie & Mulder, 2020; Haartsen & Thissen, 2014; Lundholm et al., 2004; Niedomysl & Amcoff, 2011; Thomas et al., 2019). Our findings reinforce the notion from earlier studies that living close to family deters migration, and adds to this notion that ties to family and friends are also widely reported as constraints to migration. Important to note is that we could observe these constraints while taking into account information about the actual location of the social network, which is frequently lacking from survey data.

Although we find evidence for associations between reporting specific constraints to migration and the respondents’ background characteristics, several of our results did not support the expected hypotheses. For example, we found little variation in the likelihood of reporting ties to family and friends according to people’s background characteristics. We also found little variation according to people’s household composition in mentioning any of the constraint-categories. Potentially, these findings are specific to the Spanish context, as family solidarity is particularly strong in Spain (Reher, 1998). In other contexts, ties to family and friends might be more relevant when needs appear, which might lead to more differentiation by background characteristics. In order to gain a better understanding of the differences in perceived migration constraints across cultural contexts, it is necessary to explore these perceptions in different countries.

We also failed to find evidence of differences in reporting any of the constraint categories according to the respondents’ occupation and educational achievement. Potentially, the omitted information about homeownership and income could have provided additional insights here. Nevertheless, the lack of differentiation is remarkable given that occupation and education are consistently found to be important determinants of migration. When identifying such apparent inconsistencies between self-reported constraints and observed migration or staying behaviour, we should be careful to interpret these findings as contradicting the existing literature. It might be that while reported constraints to migration are similar among educational and occupational categories, their incentives to migrate are different. Likewise, our finding that women were more likely than men to mention ties to family and friends as constraints to migration does not necessarily contradict a previous finding that highly educated Spanish women migrate more than men (González-Leonardo et al., 2020). In order to explore how self-reported constraints relate to realized (im-)mobility behaviour, future research could combine information on self-reported constraints and longitudinal residential information.

Employing a broad definition of constraints allowed for the inclusion of respondents who did not consider migrating. Their constraints to migration—including the obstacles to even forming a desire to move—have frequently been excluded from survey routings and missed by previous studies. Overall, controlling for the respondent’s migration intentions did not change our results in a substantive way. Yet, the results for each of the categories of the control variable showed that the likelihood of reporting specific constraints differs according to the respondents’ migration intentions; it thus provides important additional information about the extent to which each constraint is experienced. Notably, the results contradict the assumption that those who do not consider migrating would be less likely to report any constraints (see also: Appendix 1, Table 6). Our results further suggest that ties to the residential environment and work may be important factors that keep people from even forming a desire to migrate. Further research is needed to investigate the self-reported motivations for moving and staying depending on the stage of the decision-making process.

Self-reported constraints are important to explore in light of the observed declining migration rates and low mobility trends across the developed world. Such trends do not fit with modern stereotypes of a hypermobile society and various presumptions about the importance of internal migration for individuals and societies alike (see, for example: Cooke, 2011; Fischer, 2002). A traditionally immobile population, like Spain, offers an interesting research context to study constraints to migration. On the whole, our findings demonstrate the added value of including questions on constraints to migration in surveys, and to ask such questions not only to those who consider migrating, but also to those who intend to stay.

## Appendix 1: Operationalisation of Self-Reported Constraints to Migration

### The Open-Ended Question: Implications for Definitions

We used the answers to the open-ended question *‘¿Cuál es la razón principal por la que te sería difícil cambiar de lugar de residencia?’* (What is the main reason why it would be difficult for you to change your place of residence?) to construct our dependent variables. All respondents were asked this question, regardless of their moving intentions. Therefore, the answers to the question represent self-reported constraints to migration at all stages of the decision-making process, including considering, planning, or realising a move. Further instructions in the survey, *‘Por lugar nos referimos a la población donde resides o sus alrededores’,* imply that our definition of migration encompassed any moves away from the current town or village of residence and its surroundings, as interpreted by the respondents.

### Coding the Responses Using an Inductive Approach

The original answers to the open-ended questions were in Spanish. As one of the authors is a native of Spain, this presented no translation issues. The first read-through of the responses provided an overview of the answers and an opportunity to recode odd and non-classifiable answers (i.e.: *'vbnhtggffsi*'; '*yes*') to missing responses. We completed the coding process over three more rounds of revisions in which we labelled each constraint using an inductive approach, resulting in a primary coding scheme with keywords in English.

Our coding scheme, once aggregated, was similar to the scheme produced by Vidal and Busqueta (2020) using the same data, although ours was more specific in crucial dimensions, such as family-related codes. Our final coding scheme consisted of 30 subcategories grouped into six overarching dimensions: *1. work and economic reasons; 2. the living environment; 3. housing; 4. family; 5. friends;* and *6. other reasons;* plus a category with *missing responses.* Gillespie, Mulder and Eggleston (2021: Appendix) classified mobility motives into 64 subcategories, and later grouped them according to six larger dimensions: *1. work-related; 2. living environment; 3. housing; 4. social reasons; 5. education;* and *6. other reasons.* Even though this classification is similar to ours, there are some obvious differences. For example, Gillespie et al., (2021a, b) found that education was a prominent motive for migration that merited the construction of a separate dimension in the coding scheme, whereas the respondents in our sample rarely mentioned education as a constraint to migration. We attribute this discrepancy to differences in the nature of the questions and the samples: namely, our question was about reasons not to move and was posed to a sample of labour force participants; whereas their question was about reasons to move and was posed to anyone who migrated over 20 km. Our full coding scheme is available in table format upon request.

#### Coding Second- and Third-Order Constraints

Despite the instruction to cite the ‘main reason’, 9% of the respondents (*n* = 358) mentioned more than one constraint, and some of them mentioned three. To account for each reported constraint separately (*N* = 4289), we constructed three variables: first, second, and third constraint. This strategy was also used by Gillespie and Mulder (2020) to study primary and secondary reasons for moving.

#### Coding Ambiguously Stated Constraints

The responses differed in terms of their levels of abstraction and generalisation. For example, some respondents cited 'family' or 'money', whereas others mentioned 'being close to my mother' or 'because I am paying a mortgage'. This kind of variability is common when working with open-ended questions in computer-assisted web interviews (De Leeuw, 2002). Although this variability made it impossible for us to create specific, exhaustive, and exclusive categories, we tried to be as specific as possible when applying keywords to each response. In order to account for the uncertainty that this variability introduced into our coding process, we later grouped the responses together into subcategories and larger dimensions. Here, we used the operationalisation process of: Appendix) as an example.

### Operationalisation of the Dependent Variables

We observed that many of the self-reported constraints to migration involved sources of non-transferable capital (i.e., local ties) that have previously been shown to act as constraints to migration (David et al., 2010; Michaelides, 2011; Mulder & Malmberg, 2014; Palomares-Linares, 2018; Thomassen, 2021) and financial constraints (Landale & Guest, 1985; Sjaastad, 1962). Some examples are ‘family care obligations’, ‘a fear of change’, ‘the work location’, or feeling ‘happy here’; obstacles such as age or health; and financial limitations such as ‘income’. We thus grouped the 30 subcategories into three categories of local ties and a category for financial limitations. This yielded four dependent variables: (1) *ties to family and friends*; (2) *ties to the residential environment*; (3) *ties to work or the partner’s work*; and (4) *financial limitations*. Table 5 provides the reported frequencies per constraint category and per subcategory, and shows the associated inductive keywords.

**Table 5:** Categorisation of total responses (*N* = 4289) to the open-ended question about constraints to migration

Category	Subcategories included	Keywords	*n*
Local ties to family and friends	Family (unspecified)	Family network; family reasons	696
	Friends	Friends	269
	Closest relatives	Parents; partner; children	258
	Proximity to family	Closeness to kin; separation from family network; leaving behind family	127
	Children’s local ties	Children’s social ties; school	57
	Other social constraints	Other relatives; fear of not having social ties; pets	39
	Family care responsibilities	Family care obligations; looking after a dependent person	34
	Family attachment	Family life; family stability; family attachment	28
	*Sub-total*		*1508*
Local ties to the residential environment	Place satisfaction	Happy here, love the place; like my place	132
	Feeling settled	Stability; comfort, get used to, habit	119
	Starting from zero in another place	Fear of change; leaving the place; adaptation to another place	91
	No desire to move	I don't want to; not interested	69
	Other place related constraints	Changing lifestyle/language; missing the place of residence; distance	57
	Dwelling characteristics	House; house location; investments in the home; homeownership	48
	Quality of life	Quality of life; weather, environmental characteristics	48
	Place attachment	Attachment	45
	Structured life in place	Having a life; all here	33
	Roots	Origin, to be from; always here	29
	Amenities	Place services, place commodities, cost of living	28
	All kinds of constraints	Everything	8
	*Sub-total*		*707*
Local ties to work	Own work	Job; work conditions; permanent position	335
	Partner’s work	Partner’s permanent job; husband; boyfriend	25
	Other work-related reasons	Education; status; work location; commuting time	15
	*Sub-total*		*375*
Financial limitations	Economic limitations	Money, income, economic situation	229
	Housing costs	Mortgage payments; housing prices; moving costs	160
	Job uncertainty	Unemployed; precarious position; uncertain labour conditions; losing my job	77
	*Sub-total*		*466*
	No constraints	None; I don't have any reason in mind	112
Other answers	I don't know	I don't know	52
	Unclassified answers		40
	Health and age	Health, age	25
	*Sub-total*		*229*
*No answer*			*1004*
Total responses			4289

The total responses (*N* = 4289) differ from the total respondents in our sample (*N* = 3892) because responses that included multiple self-reported constraints are counted as multiple responses

*Source*: Survey on Attitudes and Expectations of Spatial Mobility in the Labour Force (Vidal & Busqueta, 2020), authors’ calculations

#### Ties to Family and Friends

This variable combined two dimensions of our initial coding scheme: family and friends. Some of the most frequently mentioned keywords were ‘family network', 'family reasons', and ‘friends'; and keywords related to living geographically close to family members. While these responses clearly indicated that people felt constrained by their social networks, the ambiguity in some of the answers made it impossible to know whether they were referring to family members living in their household or extended family networks. Conversely, some respondents mentioned very specific constraints, such as geographic proximity to specific kin (i.e., children, parents, and the partner). We also included responses indicating a fear of losing contact with the social network as a whole.

#### Ties to the Residential Environment

This variable combined living environment dimensions and some subcategories related to the respondents’ dwelling. Again, many responses were very generally related to place (i.e., 'I love the place'; 'I have everything here'; and expressions of 'attachment'). Based on these statements, we could infer that people felt tied to a place, but it was unclear whether they were thinking about physical characteristics only or also about the people living in that place. Other responses related to ‘feeling settled’, ‘amenities’, or ‘quality of life’. We also included responses that cited more specific characteristics of the dwelling, such as 'I like my house' and 'the surroundings of my house'.

#### Ties to Work and the Partner’s Work

This variable was constructed using the subcategories related to work from the dimension ‘work and economic reasons’. While the responses included information on the respondents’ job and employment status, they also sometimes included that of the partner. Notably, the job of the husband was mentioned much more frequently than the job of the female spouse. Other responses included the location of the job and the characteristics of the position (i.e., 'permanent job', 'well-paid job', and 'job conditions').

#### Financial Limitations

This variable combined subcategories of housing (i.e., 'paying a mortgage' and 'the housing costs of relocation') and subcategories of work and economic reasons (i.e., ‘uncertain labour conditions’ and 'no money'). Responses mentioning the lack of money and income as a limitation were the most frequent.

#### Other Responses

This category combined all the answers that could not be classified as ties to family and friends; ties to the residential environment; ties to work or the partner’s work; or financial limitations (*n* = 229). It also contained all missing responses (*n* = 1004). We are aware that missing values can be interpreted in various ways, but we included these in the reference category because people who did not report any constraint also did not mention the outcome of interest as a constraint.

It is worth noting here that open-ended survey questions are known to result in more missing responses for particular groups. This is especially likely to be the case when using computer-assisted web interviews, and missing responses are more common among individuals with lower levels of education (De Leeuw, 2002; Díaz de Rada, 2000). The descriptive findings of the missing responses (see Table 3) confirmed the presence of some biases. Singles with children, self-employed people, and manual workers were particularly likely to have missing responses. In addition, the average marginal effects (see Table 6) showed that ‘other responses’ were more common with increasing age, and among people who were living with a partner and children or other family members, and managers or directors. Thus, it seems that people who were short on time, were older, or were in a lower socio-economic position were overrepresented in this category. We took this overrepresentation into account in our interpretation of the results.

**Table 6:** Multinomial logistic regression of the most important constraint: average marginal effects and standard errors (N = 3,892)

	Other responses			Ties to family and friends			Ties to the residential environment			Ties to work			Financial limitations		
	d*y*/d*x*	SE	*P* >|*z*|	d*y*/d*x*	SE	*P* >|*z*|	d*y*/d*x*	SE	*P* >|*z*|	d*y*/d*x*	SE	*P* >|*z*|	d*y*/d*x*	SE	*P* >|*z*|
Women (ref: men)	− 0.006	0.015	0.694	0.056	0.015	0.000	− 0.003	0.012	0.794	− 0.006	0.009	0.493	− 0.041	0.010	0.000
Age (in years)	− 0.001	0.001	0.104	− 0.002	0.001	0.032	0.001	0.001	0.103	0.001	0.001	0.011	0.001	0.001	0.183
Household (ref: Single without children)															
Single with children	0.019	0.033	0.552	0.015	0.032	0.629	− 0.017	0.023	0.455	− 0.002	0.019	0.928	− 0.016	0.022	0.459
Partner without children	− 0.002	0.026	0.937	− 0.029	0.025	0.240	0.047	0.020	0.021	0.005	0.015	0.752	− 0.020	0.017	0.236
Partner with children	− 0.056	0.024	0.019	0.030	0.024	0.202	0.042	0.018	0.022	− 0.002	0.014	0.874	− 0.014	0.016	0.379
Resident family members (ref: no)	− 0.051	0.022	0.022	0.034	0.024	0.162	0.012	0.021	0.575	− 0.020	0.013	0.131	0.025	0.017	0.124
All or most of social network members live close by (ref: no)	− 0.003	0.019	0.853	0.070	0.018	0.000	− 0.023	0.016	0.140	− 0.026	0.012	0.025	− 0.017	0.013	0.183
Employment status (ref: permanent contract)															
Temporary contract	− 0.018	0.021	0.409	0.008	0.022	0.703	0.011	0.018	0.542	− 0.026	0.013	0.036	0.025	0.016	0.110
Self-employed and entrepreneurs	0.094	0.025	0.000	− 0.099	0.022	0.000	0.048	0.021	0.022	− 0.018	0.014	0.217	− 0.025	0.014	0.075
Unemployed	− 0.005	0.025	0.845	0.004	0.025	0.871	− 0.005	0.020	0.810	− 0.046	0.012	0.000	0.051	0.019	0.007
Occupation (ref: professional)															
Manual workers	0.087	0.022	0.000	− 0.045	0.022	0.042	− 0.002	0.018	0.906	− 0.014	0.013	0.283	− 0.025	0.015	0.095
Commercial, service, and administrative sector	0.017	0.021	0.417	− 0.006	0.022	0.802	− 0.010	0.017	0.547	− 0.001	0.013	0.967	0.000	0.016	0.975
Managers and directors	0.072	0.023	0.002	− 0.033	0.023	0.163	0.007	0.019	0.713	− 0.023	0.013	0.087	− 0.023	0.016	0.148
Never worked before	0.018	0.051	0.718	− 0.157	0.043	0.000	0.013	0.045	0.766	0.157	0.057	0.006	− 0.032	0.029	0.275
University education (ref: no)	0.017	0.016	0.278	0.002	0.016	0.913	− 0.006	0.013	0.646	− 0.008	0.009	0.414	− 0.005	0.011	0.622
Relationship to birthplace (ref: living in the birthplace)															
Not living in the birthplace	− 0.021	0.019	0.278	0.021	0.020	0.285	− 0.038	0.015	0.010	0.026	0.012	0.029	0.012	0.013	0.371
International migrant	0.035	0.031	0.249	− 0.067	0.029	0.019	− 0.036	0.023	0.113	0.025	0.019	0.186	0.043	0.022	0.052
Migration intention (ref: not considering to migrate)															
Considering to migrate	− 0.029	0.018	0.108	0.091	0.019	0.000	− 0.126	0.013	0.000	− 0.011	0.011	0.322	0.075	0.013	0.000
Considering and planning to migrate	0.035	0.020	0.085	0.008	0.019	0.670	− 0.101	0.015	0.000	− 0.007	0.012	0.549	0.065	0.014	0.000
Geographic region of residence (ref: North-West)															
East	− 0.035	0.027	0.188	− 0.008	0.027	0.777	0.017	0.021	0.417	0.004	0.016	0.804	0.022	0.017	0.193
South	− 0.035	0.024	0.139	0.001	0.024	0.959	− 0.001	0.018	0.960	0.004	0.014	0.795	0.031	0.015	0.034
Central	− 0.039	0.026	0.135	− 0.006	0.026	0.820	0.020	0.020	0.330	− 0.013	0.015	0.363	0.038	0.017	0.024
Metropolitan area: Madrid	− 0.056	0.026	0.035	− 0.034	0.026	0.193	0.006	0.021	0.768	0.013	0.016	0.436	0.071	0.019	0.000
Metropolitan area: Barcelona	0.012	0.028	0.674	− 0.091	0.026	0.000	0.036	0.022	0.106	0.001	0.016	0.964	0.043	0.018	0.016

‘Other responses’ category includes those respondents who did not mention any constraint

*Source*: Survey on Attitudes and Expectations of Spatial Mobility in the Labour Force (Vidal & Busqueta, 2020), authors’ calculations

#### Inclusion and Exclusion of Specific Subcategories

Responses such as those expressing 'no desire to move' were more difficult to categorise. An explorative analysis of this subcategory using the independent variables in the models revealed that individuals who did not want to move were closest to the people who mentioned place attachment or place happiness, and they were less similar to the people who left the question blank or who said 'I don't know'. Therefore, the ‘no desire to move’ subcategory was included as a tie to the residential environment. Likewise, we evaluated mentioning unemployment as a constraint by including and excluding it in the models, and eventually included it as a financial constraint. While mentioning unemployment as a constraint could be regarded as a subcategory of work, our findings indicated that feelings of financial uncertainty and not the ties to work were the driving force. To check the validity of the operationalisation, we ran models excluding and including the subcategory in doubt as a sensitivity check. The results were not substantially different from our main results (results are available upon request).

## Footnotes

1 Data collection was before the Covid-19 pandemic. A replication of the analyses using information collected at the time of or after the pandemic could result in different findings.

2 Spanish wording of the open-ended question: *‘¿Cuál es la razón principal por la que te sería difícil cambiar de lugar de residencia?’.* Spanish wording of further instructions: *‘por lugar nos referimos a la población donde resides o sus alrededores’*.
